# Identifying Subgroups with Differential Responses to Amiodarone among Cardiac Arrest Patients with a Shockable Rhythm at Hospital Arrival using the Machine Learning Approach

**DOI:** 10.31083/j.rcm2507268

**Published:** 2024-07-22

**Authors:** Ryo Emoto, Mitsuaki Nishikimi, Kazuya Kikutani, Junki Ishii, Shinichiro Ohshimo, Shigeyuki Matsui, Nobuaki Shime

**Affiliations:** ^1^Department of Biostatistics, Nagoya University Graduate School of Medicine, 466-8550 Nagoya, Japan; ^2^Department of Emergency and Critical Care Medicine, Graduate School of Biomedical and Health Sciences, Hiroshima University, 739-0046 Hiroshima, Japan

**Keywords:** out-of-hospital cardiac arrest, amiodarone, shockable rhythm, machine learning, differential responsiveness

## Abstract

**Background:**

There are few reports of studies on the differential effects of amiodarone among out-of-hospital cardiac arrest (OHCA) patients with a shockable rhythm at hospital arrival. The present study aimed to investigate the clinical heterogeneity of OHCA patients with a shockable rhythm upon hospital arrival and to identify subgroups with differential responses to amiodarone, using a machine learning approach.

**Methods:**

We used the Japanese nationwide OHCA registry of the Japanese Association for Acute Medicine for this study; data from OHCA patients with a shockable rhythm at hospital arrival were included in the analyses. The primary outcome was a favorable neurological outcome at 30 days. We developed a scoring system by the weighting method with logistic likelihood loss to identify patient subgroups showing differential effects of amiodarone from the point of view of the neurological outcome and survival at 30 days.

**Results:**

Among the 68,111 cases of OHCA in the registry, the data of 2333 OHCA patients with an initial shockable rhythm at hospital arrival were analyzed. The developed score identified higher age, longer interval between the call to the emergency medical service and hospital arrival, absence of a “witness”, no defibrillation prior to hospital arrival, hypothermia at hospital arrival, and pre-hospital epinephrine administration as variables that were significantly associated with a beneficial effect of amiodarone. Based on the results of the developed scoring system, 47% (1107/2333) of the patients were considered to greatly benefit from amiodarone administration, whereas 53% (1226/2333) of patients were considered to not benefit from amiodarone administration. The effect of amiodarone on the neurological outcome at 30 days varied significantly among the subgroups identified by the developed score (${\mathrm{OR}}_{\text{interaction}}$: 1.07 [95% confidence interval (CI): 0.99–1.13], *p* = 0.005).

**Conclusions:**

We successfully developed a model that could discriminate between OHCA patients with an initial shockable rhythm at hospital arrival who would benefit or not benefit from the administration of amiodarone in terms of the neurological outcome at 30 days. There was clinical heterogeneity among OHCA patients with a shockable rhythm in terms of their response to amiodarone.

## 1. Introduction

Amiodarone, a Vaughan Williams class III antiarrhythmic drug, has been widely used in the field of resuscitation science, especially for patients with out-of-hospital cardiac arrest (OHCA) [1]. The Japanese national guidelines recommend the use of amiodarone for OHCA patients with a shockable rhythm upon hospital arrival who are unresponsive to defibrillation with a weak recommendation [2, 3], based on reports from randomized clinical trials (RCTs) of the beneficial effect of amiodarone administration on the survival of these patients until hospital admission [4, 5]. However, a recent RCT also showed that amiodarone administration for OHCA patients had no effect on the rate of survival at the point of hospital discharge as compared with placebo [6], which suggests that the beneficial effect of amiodarone in patients with OHCA needs further evaluation.

The beneficial effect of amiodarone for improving the neurological outcome is even less clear. Besides the absence of any RCTs showing the beneficial effect of amiodarone for obtaining a favorable neurological outcome at 30 days, basic research has indicated the possibility that amiodarone can exacerbate brain injury after cardiac arrest via its effect of causing intracellular sodium accumulation [7]. On the other hand, amiodarone could also exert beneficial effects by alleviating the hemodynamic instability in OHCA patients through its effect as an antiarrhythmic drug.

Considering the report from a previous RCT that amiodarone administration improved the survival rate only in OHCA patients with bystander-witnessed cardiac arrest (CA) [6], we hypothesized that the effectiveness of amiodarone may differ among subgroups of OHCA patients with a shockable rhythm at hospital arrival. The aim of the present study was to explore the clinical heterogeneity of OHCA patients with a shockable rhythm upon hospital arrival and to identify subgroups who exhibit differential responses to amiodarone using a machine learning approach.

## 2. Materials and Methods

### 2.1 Study Design

This study was a retrospective, observational study conducted using data from an OHCA registry of the Japanese Association for Acute Medicine (JAAM-OHCA), which is a national, prospective, multicenter registry of OHCA patients transported to emergency and critical care medical centers or hospitals with an emergency care department across Japan (a total of 137 institutions). The design of the registry and the data collection method are described in detail in previous reports [8]. In brief, emergency medical services (EMS) personnel collect pre-hospital data based on the Utstein-style template [9], and physicians at the participant institutions collect in-hospital data, including the presumed etiology of the OHCA, along with the treatments employed and patient outcomes. This registry includes the data of OHCA patients entered onto the registry between June 2014 and December 2020. This study was conducted with the approval of the Institutional Review Boards of all the participant institutions; the Institutional Review Boards of all institutions waived the requirement for obtaining for informed patient consent from the study participants to ensure participant anonymity, as stipulated in the Japanese government guidelines.

### 2.2 Subjects

Adult OHCA patients with a shockable rhythm at hospital arrival, who are candidates for the administration of amiodarone in the hospital setting, were included in this study. It should be noted that the administration of amiodarone by EMS staff in the pre-hospital setting is not allowed by law. The patients were excluded if they were <18 years old, had received other antiarrhythmic drugs, could not be connected with the pre-hospital Utstein data, or had any missing data, including for the outcome and any other variables used for developing the score.

### 2.3 Candidate Variables

The variables for developing the score were limited to those that would be available prior to hospital arrival, including the baseline patient characteristics and contents of treatments in the pre-hospital setting, because the aim was to develop a score that could be calculated upon hospital arrival. We decided to set 34 ℃ as the cutoff point for hypothermia in our study because many studies investigated the metabolism, effect, or disposition of drugs at a temperature of ≤34 ℃, which was defined as hypothermia [10, 11, 12]. In some patients, the body temperature was unmeasurable at hospital arrival, and we regarded that the body temperature in these patients was too low to measure. Targeted temperature management (TTM) at 32 ℃–36 ℃ is considered for OHCA patients who are in a coma (Glasgow Coma Scale ≤8) after return of spontaneous circulation (ROSC), according to the recommendation of the Japanese resuscitation guidelines [3]. We defined mild therapeutic hypothermia as TTM at 32 ℃–34 ℃. The protocol for TTM adopted for the patients in our study, including setting the core temperature, depended on the protocol followed at each participating hospital. However, in most patients, it was maintained for 24 h, unless it had to be interrupted because of the occurrence of complications, such as hemodynamic instability. Rewarming was performed gradually over a period of at least 24–72 h.

### 2.4 Outcomes

The primary outcome was neurological outcome at 30 days, defined based on the Cerebral Performance Category (CPC): CPC 1, full recovery; CPC 2, moderate disability; CPC 3, severe disability; CPC 4, coma or vegetative state; CPC 5, died. Categories 1–2 were considered as representing a favorable neurological outcome, and categories 3–5 represented a poor neurological outcome [13]. We also evaluated the survival rate at 30 days as a secondary outcome.

### 2.5 Statistical Analysis

We estimated the individual treatment effects (ITEs) for a favorable neurological outcome at 30 days for each patient using the propensity score weighting method within a general framework for subgroup identification [14]. To prevent overfitting and ensure accurate estimation of the predictive performance of our scoring system, we integrated machine learning techniques. This approach included implementation of the cross-validation (CV) and regularization methods, which are essential in maintaining the model’s generalizability and robustness. Specifically, we first estimated the propensity score using a logistic regression with a lasso penalty. We then derived a linear personalized benefit scoring system by minimizing the lasso-penalized loss function for patient data weighted by the propensity score. Here, for the loss function, we adopted a logistic likelihood loss with modified covariates and determined the penalty parameter by CV in each of the two model-fitting stages. If the score was ≥0, a beneficial effect of amiodarone could be expected; otherwise, a harmful effect of amiodarone could be expected. And in this study, we identified subgroups, based on the patients’ scores, who showed differential effects of amiodarone.

We employed a nested CV for internal validation of the derived scoring system. Throughout the outer CV loop, a cross-validated score was calculated for each patient based on the models optimized in the inner CV loop. Based on the cross-validated score, all patients were divided into two subgroups using a cutoff point of 0. To evaluate the difference in the amiodarone efficacy among the subgroups, a point estimate of the adjusted odds ratio (OR) representing the subgroup-by-amiodarone interaction was obtained using a multivariate logistic model adjusted for other treatment variables in addition to the baseline covariates used to derive the score. For a robust inference, a non-parametric bootstrap method with 10,000 repetitions was employed to estimate the 95% confidence interval for the OR of the interaction term. We also obtained a one-sided *p* value to test the interaction term, assuming beneficial effects of amiodarone in the subgroup with positive score values, using a permutation test, where all the model fitting stages were performed from scratch after permutations for amiodarone treatment. Additionally, the impact of the interaction on survival at 30 days was evaluated using the same methods.

*p* < 0.05 was regarded statistically significant. All the statistical analyses were conducted using the R, version 4.1.1 (https://www.R-project.org/). We used the “glmnet” package [15, 16] for the lasso regression in R.

## 3. Results

**Fig. 1.**
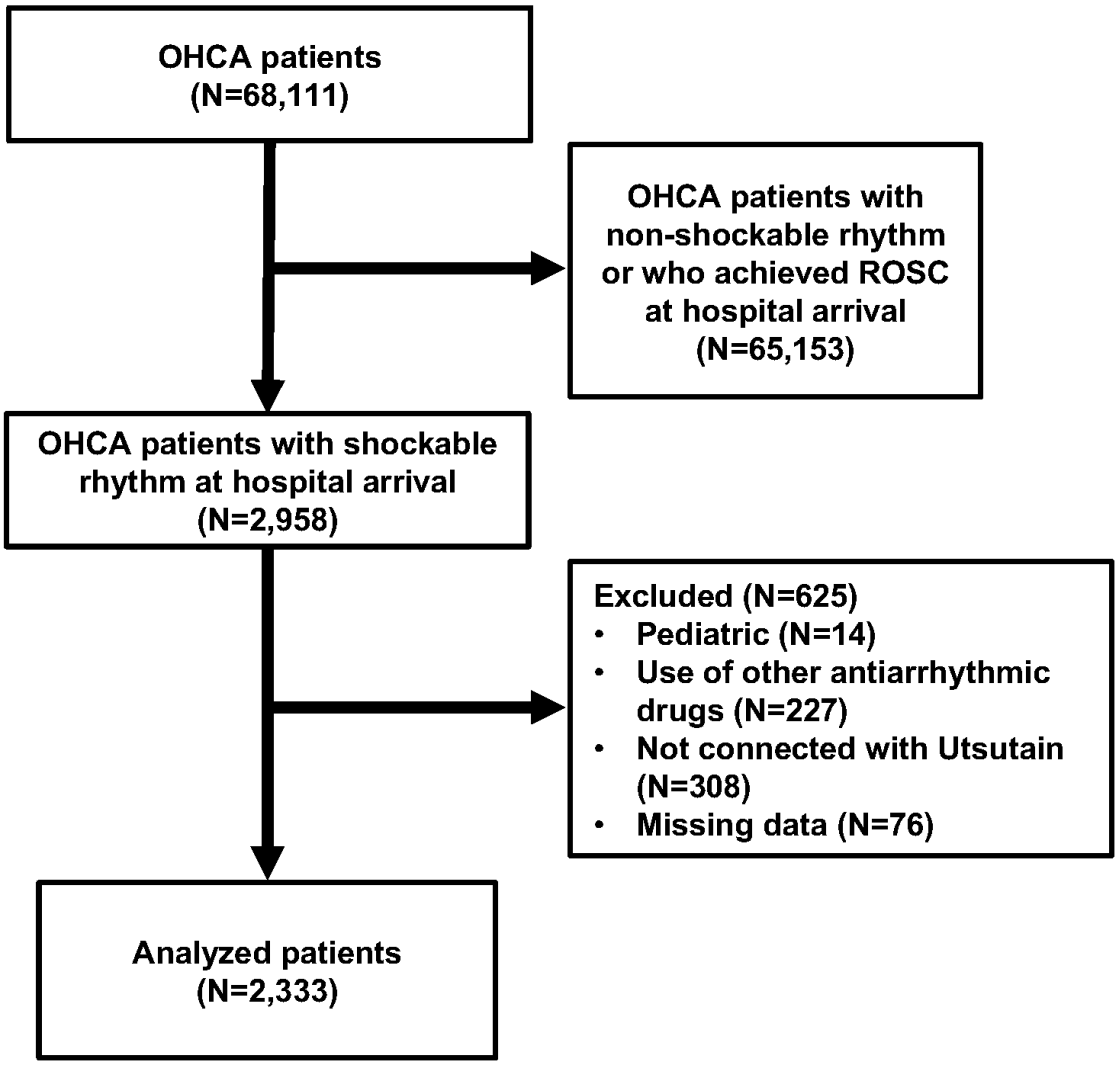
**Patient flow.** OHCA, out-of-hospital cardiac arrest; ROSC, return of spontaneous circulation.

The patient flow diagram for this study is shown in Fig. 1. Among the 68,111 registered OHCA patients in the registry, 2958 had a shockable rhythm at hospital arrival. Among these, 625 patients were excluded because they were pediatric cardiac arrest cases (n = 14), had received treatment with other antiarrhythmic drugs (n = 227), could not be connected with the pre-hospital Utstein data (n = 308), or had missing data (n = 76). Data for the remaining 2333 patients were included in the analyses.

The baseline characteristics of the patients are summarized in Table 1. Of the 2333 patients, 48.8% (1138/2333) had received amiodarone (amiodarone [+] group), while 51.2% (1195/2333) had not (amiodarone [–] group). The survival rate at 30 days was 24.4% (277/1138) in the amiodarone (+) group and 21.5% (257/1195) in the amiodarone (–) group. The proportion of patients with a favorable neurological outcome at 30 days was 13.9% (158/1138) in the amiodarone (+) group and 14.8% (177/1195) in the amiodarone (–) group.

**Table 1.** **Baseline characteristics of all subjects**.

		Amiodarone (+)	Amiodarone (–)	Overall
		(n = 1138)	(n = 1195)	(n = 2333)
Age, yrs, median (interquartile range)		65.0 (53.0–80.0)	70.0 (57.0–73.0)	67.0 (55.0–77.0)
Sex, male, n (%)		936 (82.2)	894 (74.8)	1830 (78.4)
Time from call to EMS until hospital arrival, min, median (interquartile range)		28.0 (22.0–36.0)	29.0 (22.0–34.0)	28.0 (22.0–35.0)
Cause for CA, trauma, n (%)		28 (2.5)	129 (10.8)	157 (6.7)
Witness, yes, n (%)		833 (73.2)	775 (64.9)	1608 (68.9)
Bystander, yes, n (%)		576 (50.6)	575 (48.1)	1151 (49.3)
Defibrillation, n (%)				
	0 times	413 (36.3)	634 (53.1)	1047 (44.9)
	1 or 2 times	240 (21.1)	287 (24.0)	527 (22.6)
	≥3 times	485 (42.6)	274 (22.9)	759 (32.5)
Hypothermia at hospital arrival, yes, n (%)		554 (48.7)	599 (50.1)	1153 (49.4)
Pre-hospital epinephrine administration, yes, n (%)		528 (46.4)	541 (45.3)	1069 (45.8)
ECPR, n (%)		636 (55.9)	301 (25.2)	937 (40.2)
PCI, n (%)		351 (30.8)	179 (15.0)	530 (22.7)
MTH, n (%)		270 (23.7)	161 (13.5)	431 (18.5)
Survival at 30 days, n (%)		277 (24.3)	257 (21.5)	534 (22.9)
Good neurological outcome (CPC ≤2) at 30 days, n (%)		158 (13.9)	177 (14.8)	335 (14.4)

Data are presented as the median and interquartile range (25–75% percentile) or as absolute frequencies with percentages. EMS, emergency medical services; CA, cardiac arrest; PCI, percutaneous coronary intervention; MTH, mild therapeutic hypothermia; CPC, Cerebral Performance Category; ECPR, extracorporeal cardiopulmonary resuscitation.

**Fig. 2.**
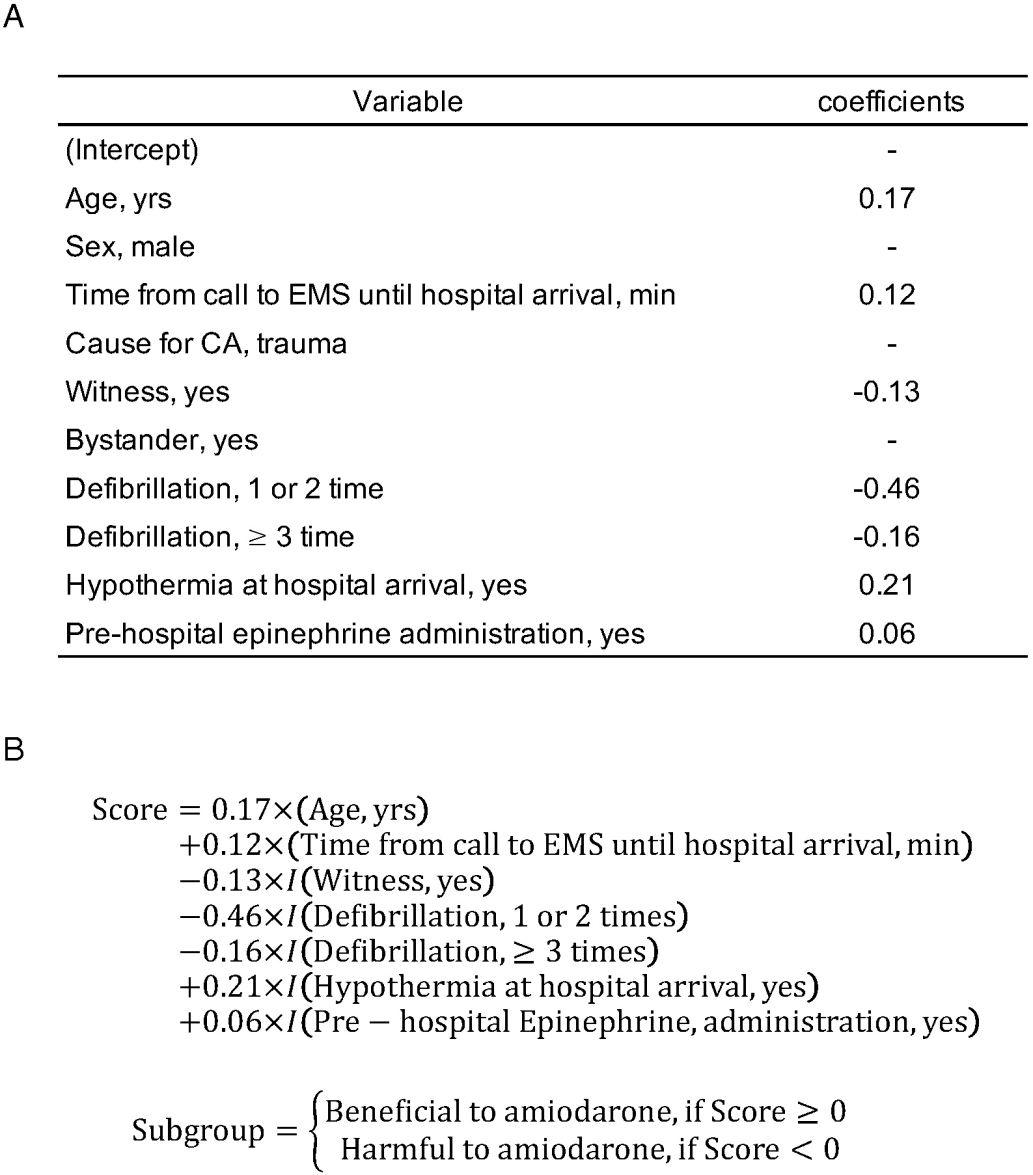
**Developed scoring system for classification of OHCA patients into subgroups with differential effects of amiodarone to the neurological outcome at 30 days.** We developed the scoring system for the classification of OHCA patients into subgroups with differential effects of amiodarone on the neurological outcome at 30 days. By using coefficients, as shown in (A), the score can be calculated as shown in (B). In patients with a score ≥0, a beneficial effect of amiodarone could be expected, while in patients with a score <0, no beneficial effect of amiodarone could be expected. EMS, emergency medical services; CA, cardiac arrest; OHCA, out-of-hospital cardiac arrest.

Then, we developed a scoring system for estimating the individualized treatment effects of amiodarone. Several factors were estimated to enhance the beneficial effect of amiodarone from the point of view of obtaining a favorable neurological outcome at 30 days, as follows; higher age; a longer interval between the call to the EMS and hospital arrival; absence of any witness(es), no defibrillation prior to hospital arrival; hypothermia at hospital arrival; and pre-hospital epinephrine administration. The coefficients for each of the variables in the developed score are summarized in Fig. 2.

The distribution of the cross-validated scores among the analyzed patients in the validation analysis is summarized in **Supplementary Fig. 1**. In the subgroup in which a beneficial effect of amiodarone was predicted based on the cross-validated score, we confirmed that a higher proportion of patients showed a favorable neurological outcome and survival at 30 days in the amiodarone (+) group than in the amiodarone (–) group (favorable neurological outcome at 30 days: 8% vs. 5%; survival at 30 days: 15% vs. 9%). Furthermore, in the subgroup with no expected benefit from amiodarone administration, a lower proportion of patients showed a favorable neurological outcome and survival at 30 days in the amiodarone (+) group than in the amiodarone (–) group (favorable neurological outcome at 30 days: 16% vs. 21%; survival at 30 days: 28% vs. 30%) (**Supplementary Fig. 2**).

In the analysis based on the multivariate logistic model with subgroup-by-amiodarone interaction, we found that the subgroup judged as amiodarone beneficial (based on the cross-validated score) showed a significantly increased effect of amiodarone on a good neurological outcome in 30 days compared with the subgroup judged as amiodarone harmful (${\mathrm{OR}}_{\text{interaction}}$; 1.07 [0.99–1.13], *p* = 0.005 [one-side permutation test]) (Table 2). Furthermore, the subgroup judged as amiodarone beneficial also showed a significantly increased effect of amiodarone on 30 days survival (${\mathrm{OR}}_{\text{interaction}}$; 1.06 [0.98–1.14], *p* = 0.024) (Table 3).

**Table 2.** **Estimated odds ratios and 95% bootstrap CIs in the multivariate logistic regressions for a good neurological outcome with the subgroup identified by the score for the neurological outcome at 30 days**.

Variable	Odds ratio (95% CI)
Age, yrs	0.93 (0.91–0.95)
Sex, male	0.99 (0.96–1.02)
Time from call to EMS until hospital arrival, min	0.97 (0.95–0.99)
Cause for CA, trauma	1.01 (0.96–1.07)
Witness, yes	1.07 (1.04–1.10)
Bystander, yes	1.02 (0.99–1.04)
Defibrillation, 1 or 2 time	1.05 (1.00–1.10)
Defibrillation, ≥3 time	1.02 (0.98–1.05)
Hypothermia at hospital arrival, yes	0.95 (0.91–0.98)
Pre-hospital epinephrine administration, yes	0.91 (0.88–0.93)
ECMO, yes	0.91 (0.88–0.94)
PCI, yes	1.14 (1.09–1.20)
MTH, yes	1.17 (1.12–1.23)
Amiodarone, yes	0.93 (0.90–0.97)
Subgroup, positive	1.00 (0.94–1.06)
Interaction, Amiodarone * Subgroup	1.07 (0.99–1.13)†

†*p* value of one-side interaction test was 0.005. EMS, emergency medical services; CA, cardiac arrest; ECMO, extracorporeal cardiopulmonary resuscitation; PCI, percutaneous coronary intervention; MTH, mild therapeutic hypothermia; CI, confidence interval.

**Table 3.** **Estimated odds ratios and 95% bootstrap CIs in the multivariate logistic regressions for 30 days survival with the subgroup identified by the score for the neurological outcome at 30 days**.

Variable	Odds ratio (95% CI)
Age, yrs	0.94 (0.92–0.96)
Sex, male	0.97 (0.94–1.01)
Time from call to EMS until hospital arrival, min	0.96 (0.94–0.98)
Cause for CA, trauma	1.04 (0.97–1.11)
Witness, yes	1.09 (1.06–1.13)
Bystander, yes	1.02 (0.99–1.05)
Defibrillation, 1 or 2 time	1.05 (1.00–1.10)
Defibrillation, ≥3 time	0.99 (0.96–1.03)
Hypothermia at hospital arrival, yes	0.94 (0.90–0.97)
Pre-hospital epinephrine administration, yes	0.91 (0.88–0.94)
ECMO, yes	0.95 (0.91–0.99)
PCI, yes	1.20 (1.14–1.27)
MTH, yes	1.34 (1.27–1.41)
Amiodarone, yes	0.94 (0.90–0.99)
Subgroup, positive	0.99 (0.93–1.06)
Interaction, Amiodarone * Subgroup	1.06 (0.98–1.14)†

†*p* value of one-side interaction test was 0.024. EMS, emergency medical services; CA, cardiac arrest; ECMO, extracorporeal cardiopulmonary resuscitation; PCI, percutaneous coronary intervention; MTH, mild therapeutic hypothermia; CI, confidence interval.

We also developed and evaluated the scoring model for estimating the effects on survival at 30 days. The factors selected for the score were the same with those for the model for neurological outcomes at 30 days, except that only the factor of cause of CA was included (**Supplementary Fig. 3**). The score distribution is shown in **Supplementary Fig. 4**, and the relationships between subgroups based on scores, actual administration of amiodarone, and survival at 30 days are presented in **Supplementary Fig. 5**. The results of the multivariate logistic regression for the subgroup-by-amiodarone interaction were consistent with those obtained for neurological outcomes (**Supplementary Tables 1,2**).

## 4. Discussion

From a national prospective database of OHCA patients, we successfully identified subgroups exhibiting differential responses to amiodarone, reflecting the heterogeneity among patients with a shockable rhythm upon hospital arrival. The developed score for estimating the individualized treatment effect of amiodarone from the viewpoint of obtaining a favorable neurological outcome at 30 days can be helpful for clinical decision-making in terms of whether amiodarone administration or other treatment options should be attempted in these patients.

In developing the score, the use of the machine learning-based methods allowed us to separate the estimation of interaction between amiodarone and the candidate variables from the estimation of the effects of prognostic factors that have a consistent impact on the outcome, regardless of the treatment. The variable selection using a lasso penalty was conducted to prevent overfitting in estimating the individualized treatment effect (ITE). As a result, we successfully identified subgroups with a high accuracy.

Surprisingly, our results showed that patients with not-witnessed cardiac arrest may derive a beneficial effect from the use of amiodarone, which was inconsistent with the results of a previous RCT [6]. Although, the primary endpoint differed between our study and the aforementioned RCT (favorable neurological outcome at 30 days in our study and survival at 30 days in the RCT), the possible reasons for the aforementioned discrepancy need to be carefully evaluated in a future study. The negative impact of the presence of defibrillation during transportation to the hospital on the beneficial effect of amiodarone is not surprising, as sustained cardiac arrest despite defibrillation may well indicate cardiac arrest refractory to pharmacological treatments, including administration of amiodarone. For such patients, other treatment options, including immediate extracorporeal cardiopulmonary resuscitation (ECPR) upon hospital arrival, should likely be prioritized over administering amiodarone, although further studies are needed to confirm this.

There were several limitations of our study. First, although our analysis based on CV successfully indicated the predictive accuracy of our scoring system for identifying patients who would benefit from amiodarone administration, further validation studies using external patient populations for external validation are warranted. Second, our scoring system may be the most useful in cases wherein ECPR can be performed at any time because this scoring system is helpful in deciding whether ECPR should be attempted without the administration of amiodarone. We need to consider the differences in the characteristics of the patients in our registry and those who might benefit greatly from our scoring system. Third, the existence of a selection bias cannot be completely excluded, because the decision to administer amiodarone and perform ECPR was left to the preference of each participating hospital. Fourth, percutaneous coronary intervention (PCI) was performed for only 22.7% of the total studied patients in our registry. The possibility of missing unknown confounding factors in the assessment of the effect of amiodarone treatment is perhaps the greatest limitation of our study. To confirm and validate this, a randomized controlled clinical trial using patient restriction or stratification based on the scoring system developed in our study would be required. Another potential limitation of using our data-driven scoring system in practice is that its interpretation may not be sufficiently clear. Further studies are needed to evaluate the pathophysiological mechanisms of the associations of the selected variables with the outcomes. Finally, although the primary outcome in our study was the neurological outcome at 30 days, as in several previous studies [17, 18], longer-term endpoints would be better for a more precise evaluation of the outcome of the patients with post–cardiac arrest syndrome [19].

## 5. Conclusions

We successfully developed a model for discriminating the subgroup of OHCA patients with a shockable rhythm upon hospital arrival who may or may not benefit from amiodarone administration in terms of obtaining a favorable neurological outcome at 30 days.

## Data Availability

The data that support the findings of this study are available from the Japanese Association for Acute Medicine but restrictions apply to the availability of these data, which were used under license for the current study, and so are not publicly available. Data are however available from the authors upon reasonable request and with permission of the Japanese Association for Acute Medicine.

## Abbreviations

OHCA, out-of-hospital cardiac arrest; RCT, randomized clinical trial; ROSC, return of spontaneous circulation; EMS, emergency medical services; CPC, Cerebral Performance Categories; ITE, individualized treatment effect; CV, cross-validation.

## Supplementary Material

Supplementary material associated with this article can be found, in the online version, at https://doi.org/10.31083/j.rcm2507268.
