# Supplementary material for: Identifying Subgroups with Differential Responses to Amiodarone among Cardiac Arrest Patients with a Shockable Rhythm at Hospital Arrival using the Machine Learning Approach
Source: Rev Cardiovasc Med. 2024 Jul 22;25(7):268. doi: 10.31083/j.rcm2507268 (PMC11317310; doi:10.31083/j.rcm2507268)
Supplement: Supplementary file 1 [file 2153-8174-25-7-268-s1.zip › Supplementary Figs.docx]

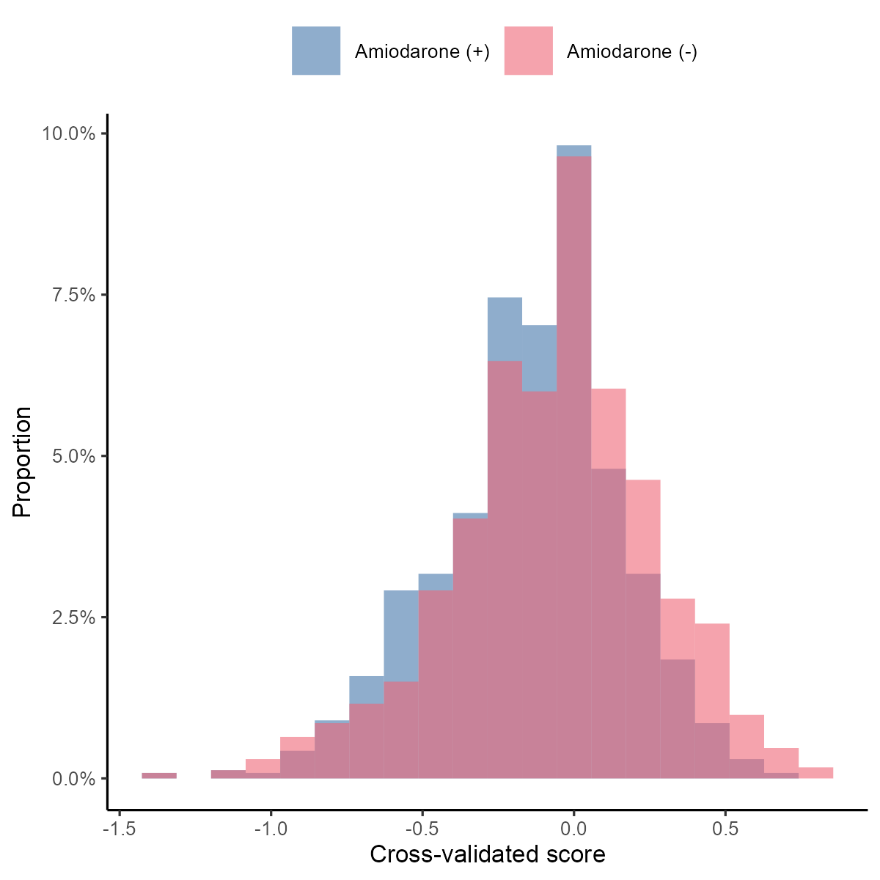


**Supplementary Fig. 1. Distribution of the cross-validated scores for the neurological outcome at 30 days in the amiodarone (+) group and amiodarone (-) group.**





**Supplementary Fig. 2. Proportion of patients with a favorable neurological outcome and survival rate at 30 days in the amiodarone (+) group and amiodarone (-) group in each subgroup classified based on the CV scores for the neurological outcome at 30 days.** *OR*, odds ratio; 95% *CI*, 95% confidence interval.

**

**

**Supplementary Fig. 3. Developed scoring system for classification of OHCA patients into subgroups with differential effects of amiodarone to the survival at 30 days.**


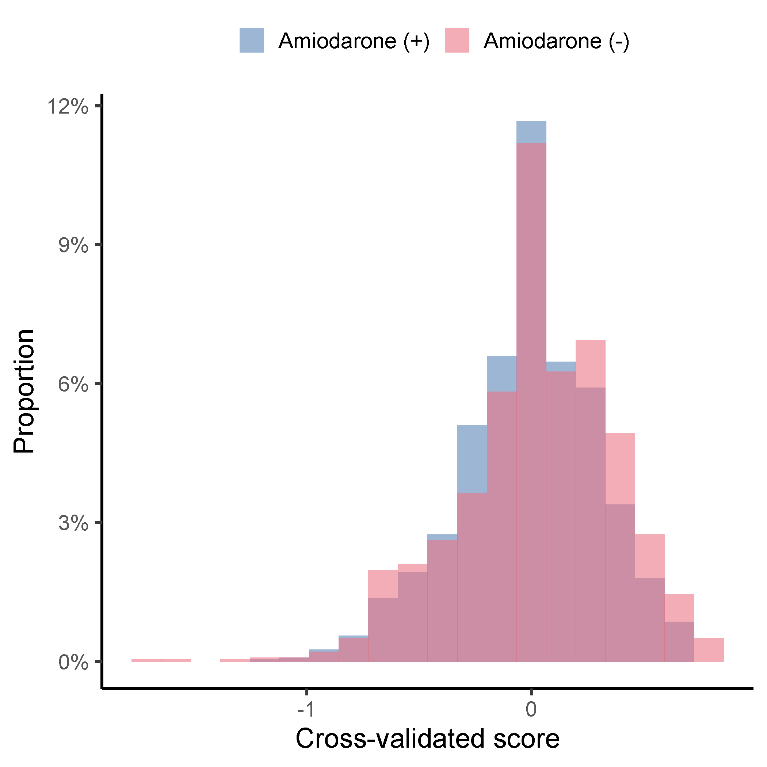


**Supplementary Fig. 4. Distribution of the cross-validated scores for the survival at 30 days in the amiodarone (+) group and amiodarone (-) group.**

**
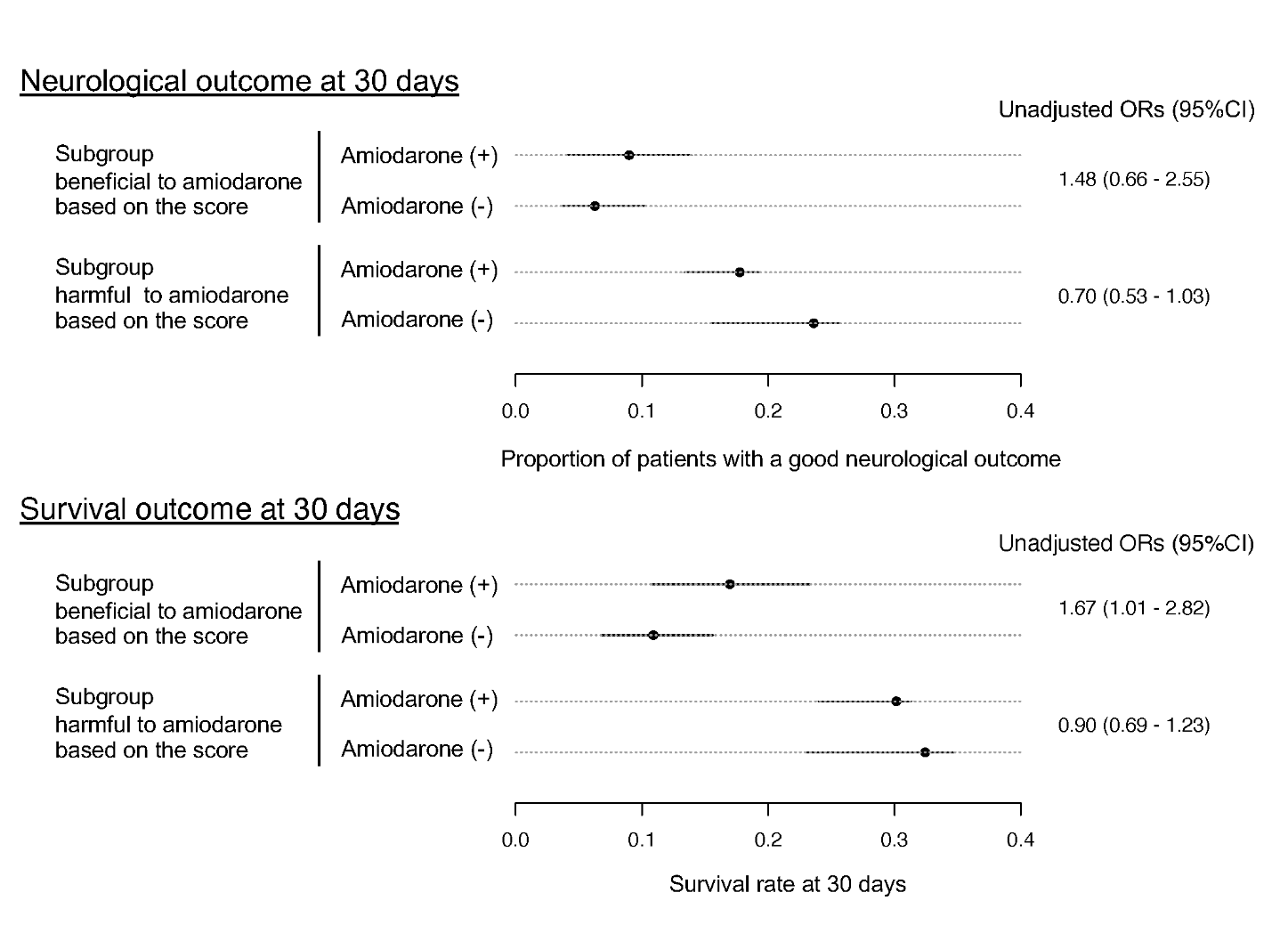
**

**Supplementary Fig. 5. Proportion of patients with a favorable neurological outcome and survival rate at 30 days in the amiodarone (+) group and amiodarone (-) group in each subgroup classified based on the CV scores for the survival at 30 days.** *OR*, odds ratio; 95% *CI*, 95% confidence interval.
