# Supplementary material for: Identifying Subgroups with Differential Responses to Amiodarone among Cardiac Arrest Patients with a Shockable Rhythm at Hospital Arrival using the Machine Learning Approach
Source: Rev Cardiovasc Med. 2024 Jul 22;25(7):268. doi: 10.31083/j.rcm2507268 (PMC11317310; doi:10.31083/j.rcm2507268)
Supplement: Supplementary file 1 [file 2153-8174-25-7-268-s1.zip › Supplementary Tables.docx]

Supplementary Table 1. Estimated odds ratios and 95% bootstrap CIs in the multivariate logistic regressions for a good neurological outcome with the subgroup identified by the score for survival at 30 days.

| Variable | Odds Ratio (95% CI) |
| --- | --- |
| Age, yrs | 0.94 (0.92-0.95) |
| Sex, male | 0.99 (0.96-1.02) |
| Time from call to EMS until hospital arrival, min | 0.98 (0.96-0.99) |
| Cause for CA, trauma | 1.01 (0.97-1.06) |
| Witness, yes | 1.06 (1.04-1.09) |
| Bystander, yes | 1.02 (0.99-1.04) |
| Defibrillation, 1 or 2 time | 1.02 (1.00-1.08) |
| Defibrillation, ≥ 3 time | 1.00 (0.98-1.04) |
| Hypothermia at hospital arrival, yes | 0.96 (0.93-0.98) |
| Pre-hospital epinephrine administration, yes | 0.91 (0.88-0.93) |
| ECMO, yes | 0.91 (0.88-0.94) |
| PCI, yes | 1.14 (1.10-1.19) |
| MTH, yes | 1.17 (1.13-1.22) |
| Amiodarone, yes | 0.92 (0.89-0.96) |
| Subgroup, positive | 0.95 (0.92-1.02) |
| Interaction, Amiodarone * Subgroup | 1.08 (1.02-1.13)† |

†P value of one-side interaction test was 0.013

EMS; emergency medical services, CA; cardiac arrest, ECMO; extracorporeal cardiopulmonary resuscitation, PCI; percutaneous coronary intervention, MTH; mild therapeutic hypothermia; CPC; Cerebral Performance Category.

Supplementary Table 2. Estimated odds ratios and 95% bootstrap CIs in the multivariate logistic regressions for 30 days survival with the subgroup identified by the score for survival at 30 days.

| Variable | Odds Ratio (95% CI) |
| --- | --- |
| Age, yrs | 0.94 (0.93-0.96) |
| Sex, male | 0.97 (0.94-1.00) |
| Time from call to EMS until hospital arrival, min | 0.96 (0.94-0.98) |
| Cause for CA, trauma | 1.04 (0.98-1.09) |
| Witness, yes | 1.09 (1.06-1.12) |
| Bystander, yes | 1.02 (0.99-1.04) |
| Defibrillation, 1 or 2 time | 1.03 (1.00-1.08) |
| Defibrillation, ≥ 3 time | 0.98 (0.96-1.02) |
| Hypothermia at hospital arrival, yes | 0.95 (0.91-0.97) |
| Pre-hospital epinephrine administration, yes | 0.91 (0.88-0.94) |
| ECMO, yes | 0.95 (0.92-0.99) |
| PCI, yes | 1.20 (1.15-1.26) |
| MTH, yes | 1.34 (1.28-1.40) |
| Amiodarone, yes | 0.93 (0.89-0.97) |
| Subgroup, positive | 0.95 (0.90-1.02) |
| Interaction, Amiodarone * Subgroup | 1.07 (1.01-1.16)† |

†P value of one-side interaction test was 0.001

EMS; emergency medical services, CA; cardiac arrest, ECMO; extracorporeal cardiopulmonary resuscitation, PCI; percutaneous coronary intervention, MTH; mild therapeutic hypothermia; CPC; Cerebral Performance Category.å
