# Supplementary figures and images for: Identifying Subgroups with Differential Responses to Amiodarone among Cardiac Arrest Patients with a Shockable Rhythm at Hospital Arrival using the Machine Learning Approach
Source: Rev Cardiovasc Med. 2024 Jul 22;25(7):268. doi: 10.31083/j.rcm2507268 (PMC11317310; doi:10.31083/j.rcm2507268)

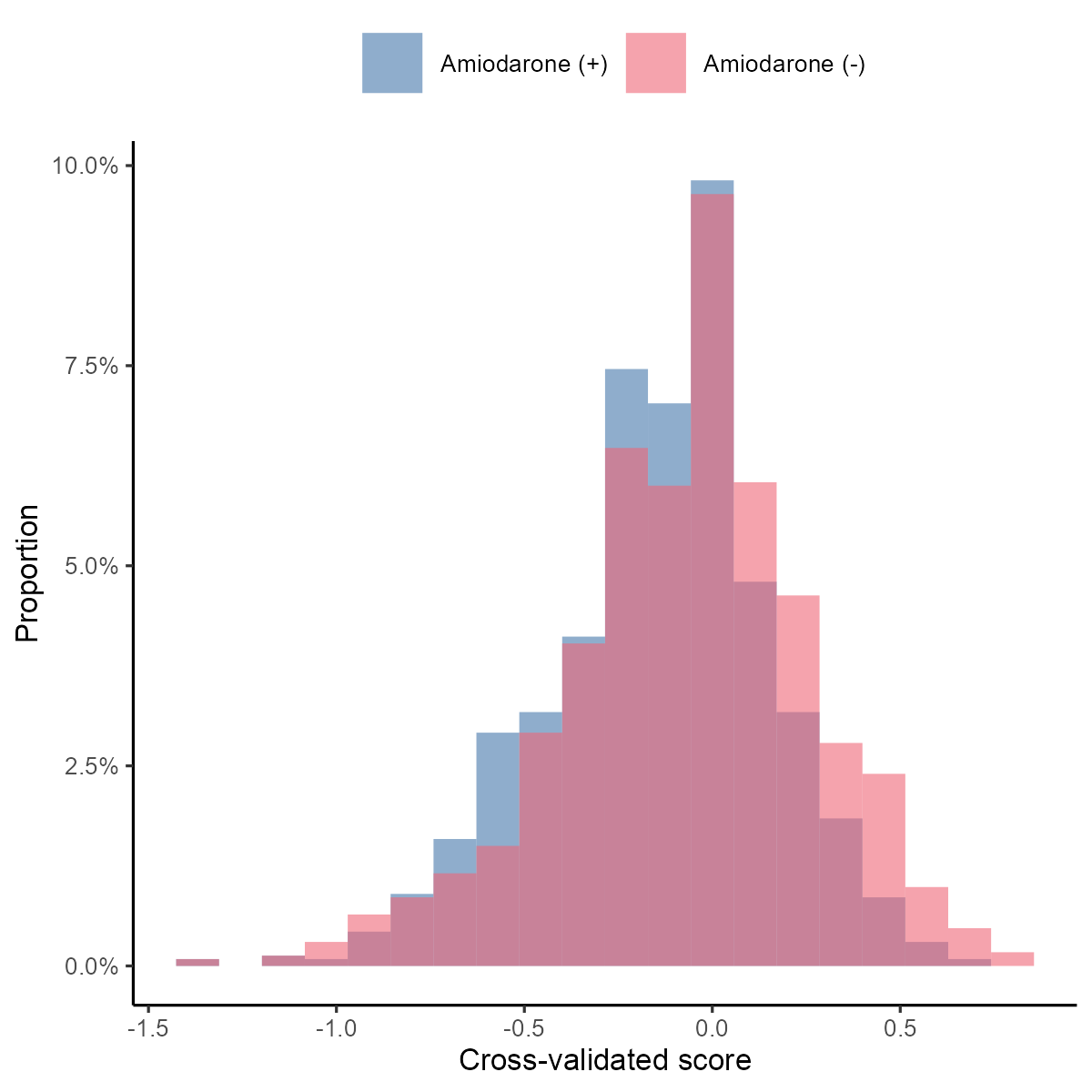

Supplement: Supplementary file 1 [file 2153-8174-25-7-268-s1.zip › Supplementary Fig. 1.tiff]

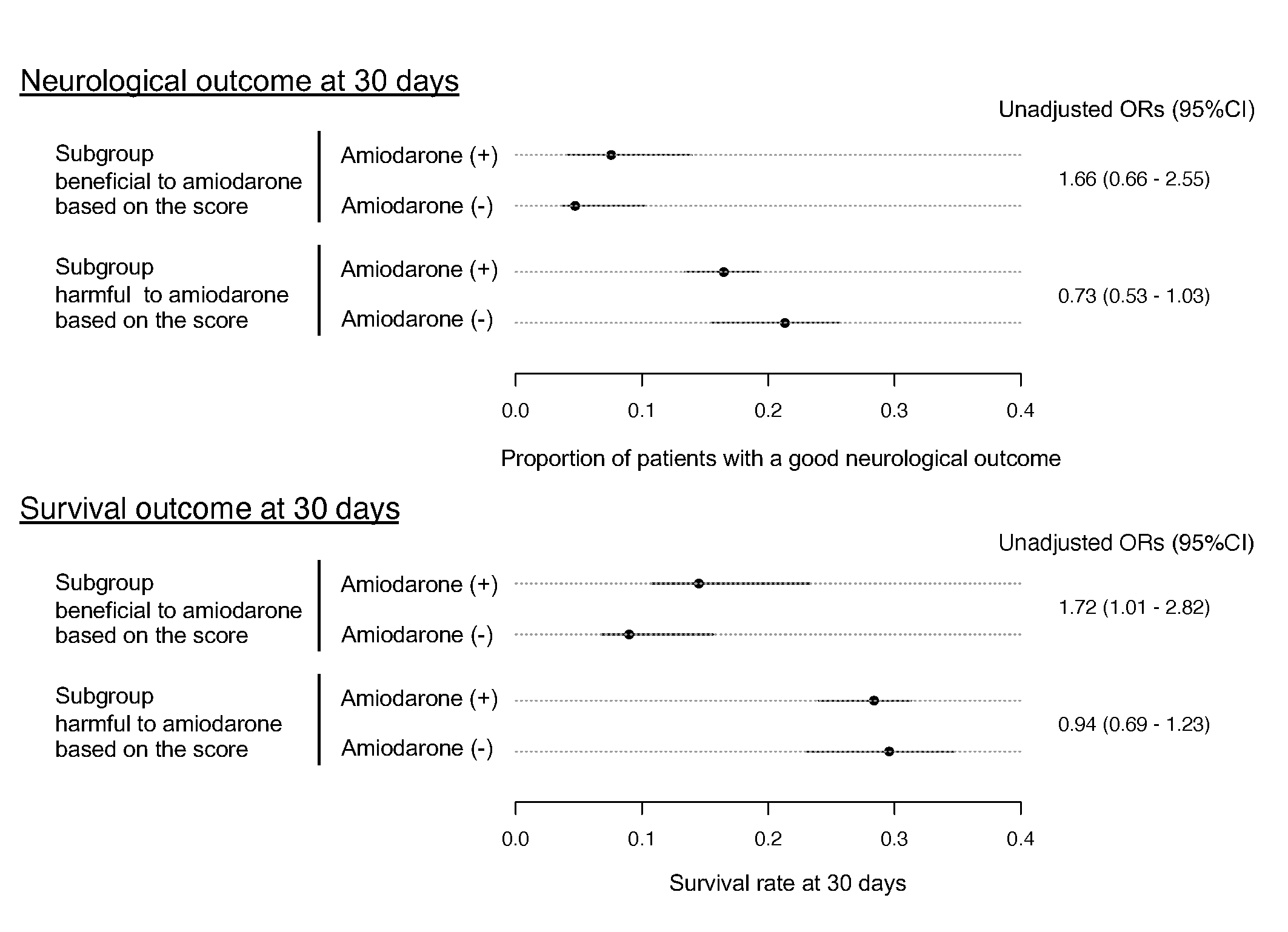

Supplement: Supplementary file 1 [file 2153-8174-25-7-268-s1.zip › Supplementary Fig. 2.tiff]

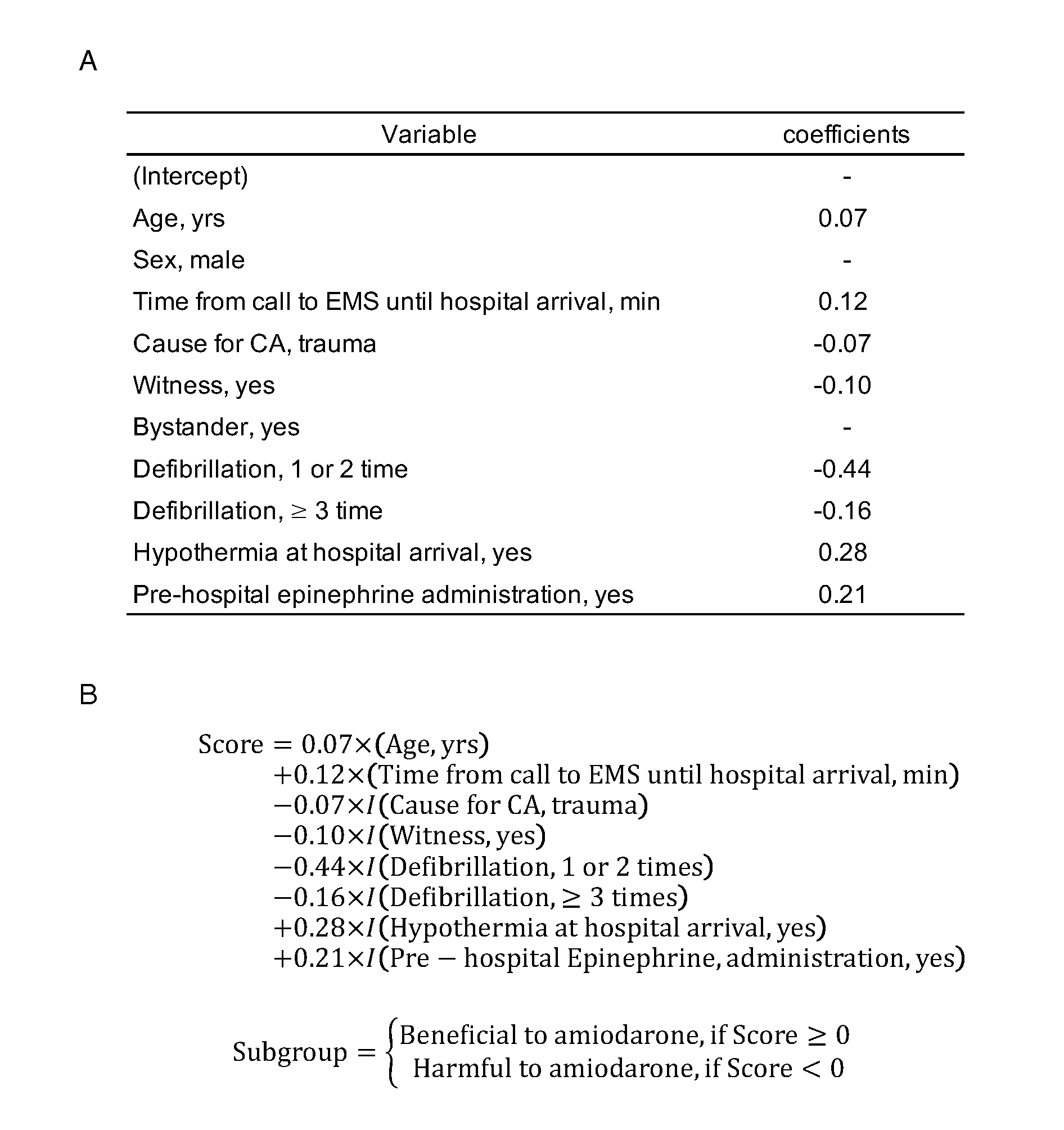

Supplement: Supplementary file 1 [file 2153-8174-25-7-268-s1.zip › Supplementary Fig. 3.tiff]

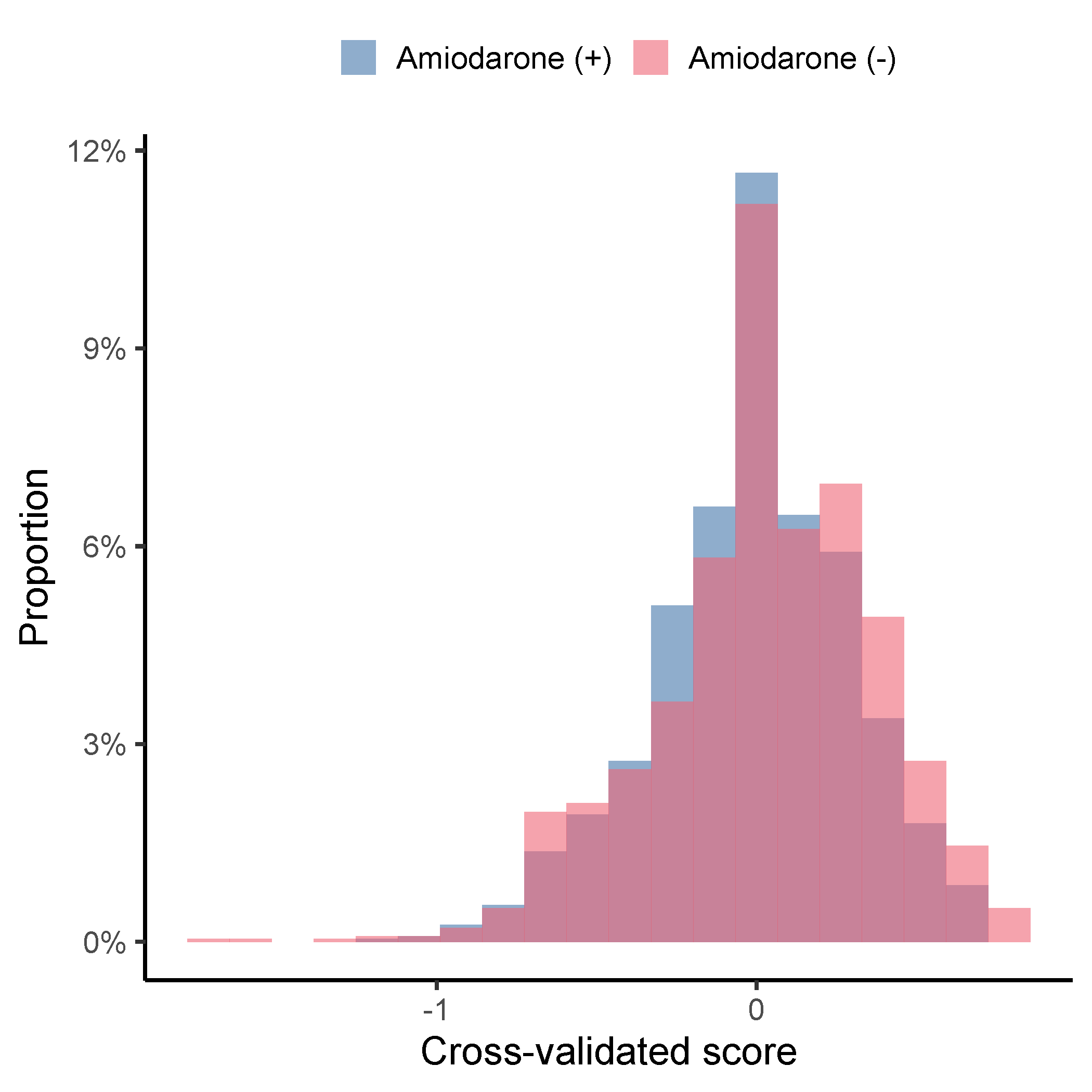

Supplement: Supplementary file 1 [file 2153-8174-25-7-268-s1.zip › Supplementary Fig. 4.tiff]

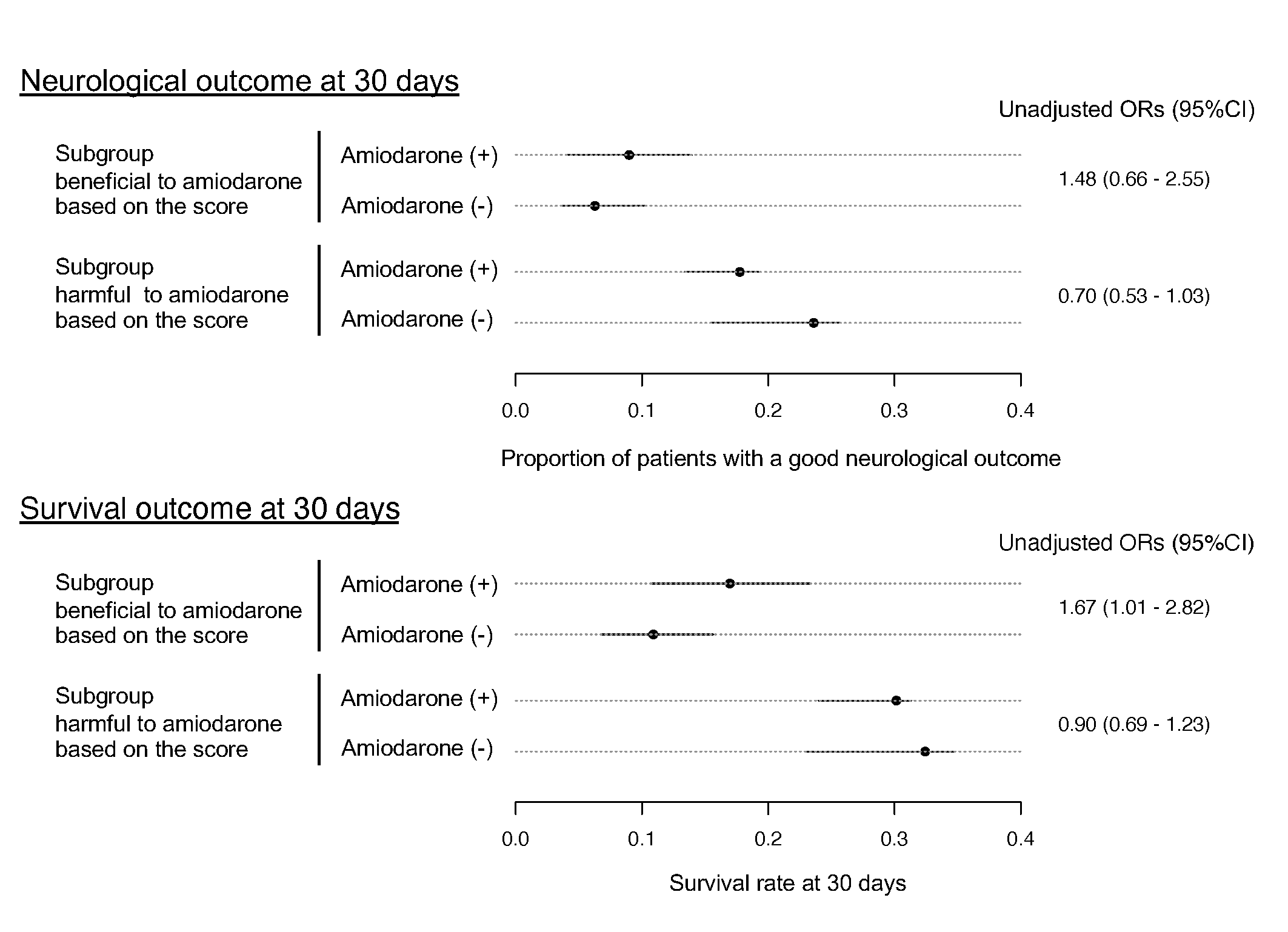

Supplement: Supplementary file 1 [file 2153-8174-25-7-268-s1.zip › Supplementary Fig. 5.tiff]
